# A locus on barley chromosome 5H affects adult plant resistance to powdery mildew

**DOI:** 10.1007/s11032-018-0858-2

**Published:** 2018-07-28

**Authors:** Sanjiv Gupta, Elysia Vassos, Beata Sznajder, Rebecca Fox, Kelvin H. P. Khoo, Robert Loughman, Kenneth J. Chalmers, Diane E. Mather

**Affiliations:** 1 Department of Primary Industries and Regional Development, 3 Baron-Hay Court, South Perth, WA 6151 Australia; 2 State Agricultural Biotechnology Centre, School of Veterinary and Life Sciences, Murdoch University, Murdoch, WA 6150 Australia; 3 School of Agriculture, Food and Wine, Waite Research Institute, PMB 1, Glen Osmond, SA 5064 Australia

**Keywords:** Adult-plant resistance, Quantitative trait loci, molecular markers, *Blumeria graminis*, *Hordeum vulgare*

## Abstract

Adult plant resistance against plant pathogens is of interest as a means to achieve durable resistance. Prior to this research, the barley lines CLE210 (from Uruguay) and Denar (from the Czech Republic) had been reported to exhibit adult-plant resistance against powdery mildew. Here, populations of doubled haploid lines from crosses of these lines with the susceptible cultivar Baudin were evaluated for powdery mildew resistance in field experiments. Using linkage maps constructed from genotyping-by-sequencing (GBS) data, it was determined that differences in resistance were largely attributable to a region on the long arm of chromosome 5H (5HL). Therefore, KASP^™^ assays were developed based on GBS tag sequences mapped on that chromosome, providing more reliable genetic maps. In each population, a large-effect QTL was mapped on 5HL. As no sequence variation was detected between CLE210 and Denar in this region of 5HL, the two sources of resistance may be identical by descent in the QTL region and carry the same resistance gene. Marker assays from the QTL region were evaluated on a panel of barley lines, providing information that breeders could use to select assays for use in marker-assisted selection.

**Electronic supplementary material:**

The online version of this article (10.1007/s11032-018-0858-2) contains supplementary material, which is available to authorized users.

## Introduction

Factors that can be used to classify and define types of resistance against plant diseases include growth stage (all-stage (seedling) resistance vs. adult-plant resistance (APR)), degree of resistance (complete resistance or immunity vs. incomplete or partial resistance), race specificity (race-specific resistance vs. non-race specific (broad-spectrum) resistance) and durability (durable vs. non-durable resistance) (Chen 2013). All-stage resistance can be detected on seedlings and remains effective throughout all growth stages. In contrast, plants with APR are susceptible as seedlings but exhibit resistance at later stages. In many cases, seedling resistance is complete, race-specific and non-durable, whilst APR is often expected to be incomplete, non-race-specific and durable.

In barley (*Hordeum vulgare L*. subsp. *vulgare*) and its wild relatives, there are many genes (reviewed by Jørgenson and Wolfe (1994) and Ames et al. (2015)) that can individually confer complete race-specific seedling resistance against powdery mildew caused by the biotrophic ascomycete fungus *Blumeria graminis* (DC.) E. O. Speer, f. sp. *hordei* emend. E. J. Marchal (anamorph *Oidium monilioides* Link). This form of resistance is generally not expected to be durable, as the pathogen can evolve to overcome it. Nevertheless, durable resistance against powdery mildew has been achieved through the use of recessive alleles of the barley gene *Mlo* (Jørgensen 1992; Büschges et al. 1997). Non-functional *mlo* alleles confer broad-spectrum resistance that is effective across growth stages. Although widely adopted, *mlo* resistance has some disadvantages and limitations. It has been reported to be associated with necrotic leaf spotting, reduced yield and increased susceptibility to several facultative fungal pathogens (see Brown and Rant (2013) for a review and McGrann et al. (2014) for a report on susceptibility to Ramularia leaf spot). In order to maintain the durability of *mlo* resistance, barley breeders have avoided deploying *mlo* in winter-habit barley. Accordingly, there is ongoing interest in the discovery of novel sources of resistance, particularly those that could offer the durability of *mlo* resistance, without its disadvantages and limitations.

Quantitative variation in the disease expression on adult plants has been used to map quantitative trait loci (QTL) for powdery mildew resistance in barley (e.g. Falak et al. 1999; Shtaya et al. 2006; Li and Zhou 2011; Silvar et al. 2011; Hickey et al. 2012). None of these loci were confirmed to be associated with APR per se. Some co-locate with known seedling resistance genes and could be associated with all-stage resistance.

**Fig. 1:**
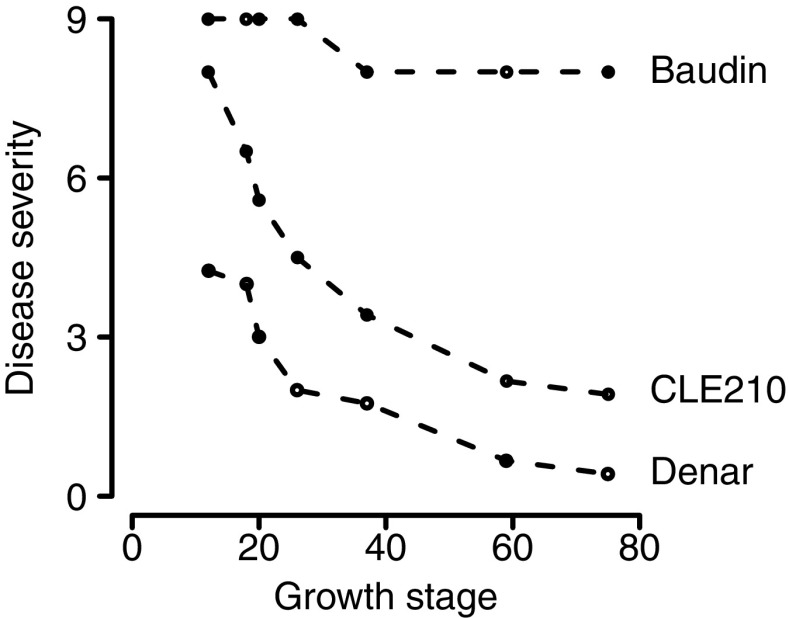
Powdery mildew disease severity on plants of Baudin, CLE210 and Denar barley, as assessed by Gupta et al. (2015) between the two-leaf growth stage (GS12; Zadoks et al. 1974) and grain filling (GS75). Disease severity was assessed on a scale from 0 (no disease) to 9 (very severe disease)

Gupta et al. (2015) discovered and characterised several sources of APR against powdery mildew of barley. Here, we report on the genetic control of powdery mildew APR in two of those lines, CLE210 and Denar. In field experiments (Gupta et al. 2015), powdery mildew at the two-leaf growth stage (GS12; Zadoks et al. 1974) was severe for CLE210 and moderate for Denar, but declined thereafter (Fig. 1). The area under the disease progress curve was 10% for CLE210 and 5% for Denar, compared to 0% for the *mlo* cultivar Alexis and 84% for the susceptible cultivar Baudin. To investigate the genetic basis for this APR, CLE210 and Denar were each crossed with Baudin. Mapping populations developed from these crosses were evaluated for resistance against powdery mildew. Genotyping-by-sequencing (Elshire et al. 2011) was used to genetically map resistance loci, anchor their positions to a barley genome assembly and provide sequence data for the design of marker assays.

## Materials and methods

### Barley material

The barley lines CLE210 (pedigree: Aphrodite (Villa/Plenum)/CLE216 (Atlas57//PriorA/Ymer/3/B6671); originating from Uruguay) and Denar (pedigree: Celechovicky Hanacky/Bavaria; originating from the Czech Republic) were each crossed with the Australian barley cultivar Baudin (pedigree: Stirling/Franklin). F_1_-derived doubled haploid (DH) lines (234 for CLE210/Baudin and 251 for Denar/Baudin) were generated using the anther culture methods described by Broughton et al. (2014). A panel of 203 other barley varieties was also used.

### Evaluation of resistance against powdery mildew

The parents and DH lines of the CLE210/Baudin and Denar/Baudin populations were grown in randomised complete block design field experiments at South Perth, WA, Australia (31° 9′S, 115° 89′E) in 2011 and 2012. Each experiment consisted of two blocks and each block contained the parents, the DH lines and several other varieties added for spatial control. The experiments were surrounded by spreader rows of the powdery mildew-susceptible parent Baudin, which had already been infected by natural inoculum of *Blumeria graminis* f. sp. *hordei* before the experiments were sown. An additional spreader row was also placed approximately in the middle of each experiment. At anthesis (Zadoks growth stage (GS 60–69)) disease severity was assessed on a scale from 0 (no signs of disease) to 9 (very severe).

In addition, the same materials were evaluated as seedlings in both 2011 and 2012. For this, 10 seeds of each parent or DH line were sown in pots to provide a ‘clump’ of seedlings. The pots were kept in a glasshouse at 18–22 °C. When the plants reached the two-leaf stage (GS 12), they were dusted with spores of powdery mildew that had been collected from the field experiments at South Perth. The virulence spectrum of prevalent isolate at South Perth location on Pallas isolines was reported by Gupta et al. (2015). The plants were assessed 8 days later using a modified version of the scale of Torp et al. (1978), ranging from 0 (no signs of disease) to 5 (very severe signs of disease).

### Genotyping by sequencing and construction of linkage maps

For genetic analysis, leaf tissue was sampled from one seedling of each parent or DH line, and genomic DNA was isolated using a phenol chloroform method (Rogowksy et al. 1991) with modifications as described by Pallotta et al. (2000). Aliquots of DNA were sent to the company Diversity Arrays Technology (Bruce, ACT, Australia) for analysis with its DArTseq genotyping-by-sequencing platform (www.diversityarrays.com/dart-application-dartseq). Within each population, segregating markers were scored as single-nucleotide polymorphisms (SNPs) (pairs of sequence tags differing only by one nucleotide) or silicoDArTs (sequence tags from one parent for which there was no exact match or single-base mismatch from the other parent).

The process of constructing genetic linkage maps involved the following steps: (1) construction of maps from the SNP marker data, (2) construction of maps with both SNP and silicoDArT markers, (3) alignment of SNP and silicoDArT maps to identify silicoDArTs markers that could be useful to improve the genomic coverage of the SNP map and (4) construction of final maps with SNP markers and selected silicoDArT markers. The map construction and diagnostics were performed with functions and workflow of the R package ASMap (Taylor and Butler 2016), which implements the MSTMap algorithm (Wu et al. 2008) and is available in the R Statistical Computing Environment (R Core Team 2016).

### Mapping of SNP markers

Prior to linkage map construction, individual markers were excluded from the genotypic data set if no polymorphism was detected, if there was extreme segregation (minor allele frequency (MAF) < 0.05) or if more than 20% of the genotypic values were missing. Similarly, individual DH lines were excluded if more than 30% (CLE210/Baudin) or 25% (Denar/Baudin) of the values were missing. The remaining genotypic data were investigated to identify pairs or groups of lines with identical or nearly identical results (sharing the same alleles for more than 90% of the markers for which data were available for all lines). For each pair or group that was discovered in this way, the individual line genotypes were collapsed to a consensus genotype (Taylor and Butler 2016).

The genotypic data were re-coded according to parental calls, with markers of unknown phase set aside. Markers showing moderate segregation distortion (0.4 ≥ MAF) were also set aside. The remaining data were used to construct skeleton linkage maps with the MSTmap algorithm (Wu et al. 2008) as implemented in R/ASMap. Markers that had been set aside due to unknown phase or moderate distortion were then ‘pushed’ into the map using the combineMap and pushCross functions of the R/ASMap package (Taylor and Butler 2016).

The sequences of the genetically mapped SNP tags were used as queries in a BLAST search (E-value cutoff, 1e−5) against version 1 of the barley genome sequence assembly (International Barley Genome Sequencing Consortium 2012) to determine the correct orientation of each linkage group and to compare genetic and physical map orders.

### Mapping of silicoDArT markers

The initial process followed for the silicoDArT marker data was the same as that followed for the SNP marker data, involving exclusion of data for monomorphic markers, markers with high rates of missing values and markers exhibiting extreme segregation distortion. Lines for which data had been excluded from the SNP map construction were excluded from the silicoDArT map construction. Consensus genotypes were determined for the same sets of lines as for the SNP map construction. The genotypic data were re-coded according to parental calls. Markers of unknown phase and markers showing moderate segregation distortion were discarded.

The remaining silicoDArT markers were pushed into the SNP-based map using the combineMap and pushCross functions of the R/ASMap package, and the genotypic data were examined for patterns of recombination. Markers with high counts of apparent crossovers in both flanking intervals were considered to be unreliable and were removed. The resulting map was aligned against the SNP-based map, and the silicoDArT tag sequences were used as queries in a BLAST search against barley genome assembly sequence data. Finally, for each region of 10 cM or longer in which no SNP markers had been mapped, one or more silicoDArT markers (about one every 5 cM) were selected and pushed into the SNP map to provide ‘curated GBS linkage maps’ for use in mapping of resistance loci.

### Statistical analysis

For analysis of the phenotypic data, each pair or group of DH lines that had been identified as genetically identical or nearly identical and for which a consensus genotype had been generated was considered to represent a single line. Statistical models were fitted using the software ASReml-R (Butler et al. 2009) available in the R Statistical Computing Environment (R Core Team 2016). For each experiment, an initial linear mixed model was fitted of the form:

$$ y= X\beta + Zu+{Z}_gg+e $$

where *y* represents the trait value (disease severity), β represents the non-genetic fixed effects with the associated design matrix *X*, *u* represents random non-genetic effects with associated design matrix *Z*, *g* represents random genetic effects with associated design matrix *Z*_g_ and *e* is the residual error. Fixed effects consisted of a type variable to differentiate between DH lines, parental lines and control lines of the field experiment. There was one genetic random intercept per line and there were non-genetic random effects related to spatial design. A two-dimensional separable autoregressive spatial model of first order (AR1 × AR1) was fitted to model natural variation amongst neighbouring plots, and a sample variogram of the residuals (Gilmour et al. 1997) was examined for indication of extraneous variation. Based on the sample variogram, additional random effects pertaining to row and/or column patterns were added to the model. The model was further scrutinised with diagnostic tools of ASReml-R for adherence to the model assumptions. This model was used in the QTL analyses.

In a similar manner, subsequent linear mixed models were constructed in which the genetic effects pertaining to individual lines were fitted as fixed effects to obtain predicted values for each line in the experiment.

### QTL analysis

Prior to QTL analysis, the genotypes for the markers and DH lines used to generate the curated GBS maps were recoded to a numeric range between − 1 and 1, with missing marker data imputed according to the method of Martinez and Curnow (1994) as implemented in the R/wgaim package (Taylor and Verbyla 2011). Initial QTL analysis was conducted using the curated GBS linkage maps. Associations between marker genotypes and trait phenotypes were analysed on a single-marker single-experiment basis by refitting linear mixed models with the fixed effects of the markers. Wald tests were used to infer the significance of the marker-trait associations, and Wald test statistics were plotted against the genetic positions of the markers to show the locations of the QTL. Significance thresholds for Wald test statistics were obtained by back-transforming from corrected *p* values at significance level α = 0.05 calculated as adjusted Bonferroni corrected *p* values (0.05/estimated effective number of independent markers (Li and Ji 2005)).

### Development and use of a KASP marker map for chromosome 5H

For chromosome 5H, on which a resistance locus was detected in each population, GBS tags were chosen for the design of uniplex assays. Primer sets were designed using Kraken^™^ software and were used to genotype each parent and DH line via KASP^™^ technology, implemented on an automated SNPLine system (LGC Limited, Teddington, UK).

One CLE210/Baudin line and two Denar/Baudin lines had to be excluded from further analysis because more than 25% of the markers were consistently genotyped as heterozygous, indicating that their DNA samples had become contaminated. Linkage maps were constructed for chromosome 5H, using the same R/ASMap workflow that had been used for construction of the GBS-based SNP map, but using only the genotypic data from the uniplex assays. QTL analysis was undertaken using the resulting KASP maps of chromosome 5H, using the same approach that had been used for genome-wide analysis, with the Bonferroni corrected *p* value set by dividing 0.05 by the number of tested markers.

The sequences of all SNP tags and silicoDArT tags were used as queries in a BLAST (Altschul et al. 1990) search (E-value cutoff 1e−5, word size 28) against the 5H pseudomolecule of the reference genome assembly of barley (Mascher et al. 2017; IBSC (2016-07-14): Pseudomolecules of the map-based reference genome assembly of barley cv. Morex. DOI:10.5447/IPK/2016/34).

## Results

### Disease severity

At the seedling stage, powdery mildew was moderate to severe on all three parents and on almost all of their doubled haploid progeny (Online Resource 1). In contrast, at the adult stage, the effects of powdery mildew were much less severe on both CLE210 and Denar than on Baudin (Table 1 and Online Resource 2) and the DH lines exhibited a wide range of ratings (Online Resource 2).

**Table 1:** Predicted values ± standard errors for powdery mildew disease severity on adult plants for CLE210, Denar, Baudin and populations of CLE210/Baudin and Denar/Baudin double haploid lines, as assessed in experiments conducted in 2011 and 2012

Experiment	Parent or population	2011	2012
CLE210/Baudin	CLE210	1.2 ± 0.4	2.2 ± 0.3
	Baudin	7.9 ± 0.1	7.3 ± 0.1
	CLE210/Baudin population	4.7 ± 1.7	4.4 ± 1.6
Denar/Baudin	Denar	1.2 ± 0.3	1.9 ± 0.3
	Baudin	8.0 ± 0.1	6.9 ± 1.4
	Denar/Baudin population	3.9 ± 1.9	3.3 ± 1.4

Disease severity was assessed on a scale from 0 (no disease) to 9 (very severe disease)

### Genome-wide mapping

Across the two populations, genotypic data were obtained for 8615 polymorphic markers: 1440 SNPs and 7175 silicoDArTs. Of these, 492 SNPs and 3379 silicoDArTs were polymorphic in both populations, 360 SNPs and 1973 silicoDArTs were polymorphic only in CLE210/Baudin and 515 SNPs and 1807 silicoDArTs were polymorphic only in Denar/Baudin.

During the construction of the SNP-based maps, some of the available polymorphic markers and some lines were excluded, mostly due to high rates of missing data: 267 markers and 10 lines for CLE210/Baudin and 136 markers and three lines for Denar/Baudin. Consensus genotypes were generated for 32 pairs of lines (22 CLE210/Baudin and 10 Denar/Baudin) and two sets of three lines (one in each population). The SNP-based maps were therefore constructed based on data for 200 CLE210/Baudin lines and 235 Denar/Baudin lines. Most of the genetically mapped SNP markers (555 of 579 for CLE210/Baudin and 821 of 869 for Denar/Baudin) could be anchored to the barley genome assembly. Of these markers, all but 18 were anchored to positions on the same chromosome as they were genetically mapped. Within chromosomes, the genetic and physical orders of the markers were similar.

In the processing of the silicoDArT data, many markers were excluded, mostly because they were closely linked with other markers (and were therefore not needed), were of unknown phase or exhibited segregation distortion. The maps constructed with both SNP and silicoDArT markers contained 4219 markers (CLE210/Baudin) and 3062 markers (Denar/Baudin). After silicoDArTs from genomic regions with sparse SNP coverage were added to the SNP-based map, the ‘curated GBS maps’ for CLE210/Baudin and Denar/Baudin had 714 markers and 982 markers, respectively (Online Resource 3).

**Fig. 2:**
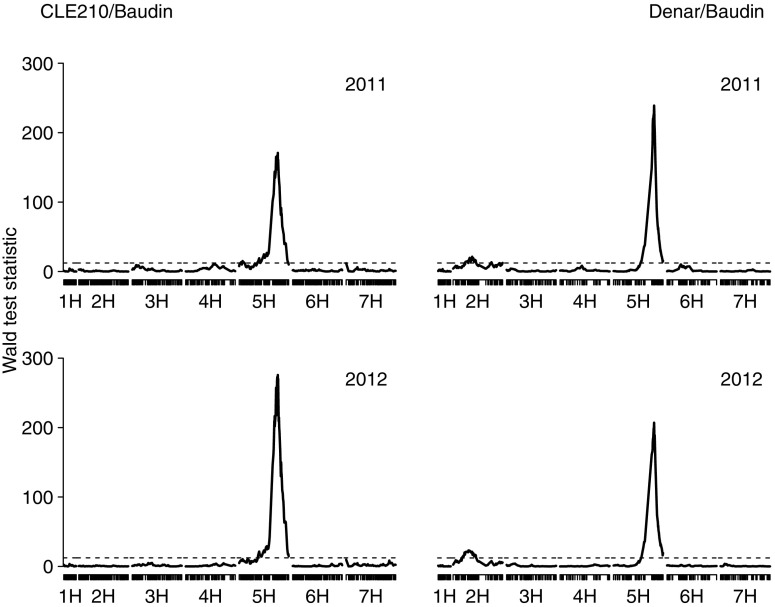
Wald test statistics for powdery mildew disease severity on adult plants in experiments conducted in 2011 (*top*) and 2012 (*bottom*), derived from single-marker analyses using genetic linkage maps of chromosome 5H based on genotyping by sequencing of the barley populations CLE210/Baudin (*left*) and Denar/Baudin (*right*). Each chromosome is depicted with the short arm on the *left* and the long arm on the *right*. The horizontal dashed lines indicate the significance thresholds at α = 0.05, corrected for multiple testing

A very highly significant QTL for disease severity in the adult stage was detected on 5HL in both populations and in both years (Fig. 2), with CLE210 and Denar as the sources of the resistance alleles. A minor-effect QTL was also detected on chromosome 2H, but only in the Denar/Baudin population, with Denar as the source of the resistance allele.

### Detailed analysis of chromosome 5H

KASP assays were designed for GBS polymorphisms that had mapped on chromosome 5H in one or both populations. Once the KASP markers had been assayed on the mapping populations, three markers were excluded (two from CLE210/Baudin and one from Denar/Baudin) because they failed to map on chromosome 5H. Of 101 KASP markers that were mapped on chromosome 5H (Online Resource 4), 95 acted as co-dominant bi-allelic SNPs whilst six acted as dominant presence-absence variants, with one of the alleles classified as a ‘null’ allele because no fluorescence was detected. The KASP-based maps of chromosome 5H were much shorter than the maps derived directly from the GBS data (Online Resource 3).

**Fig. 3:**
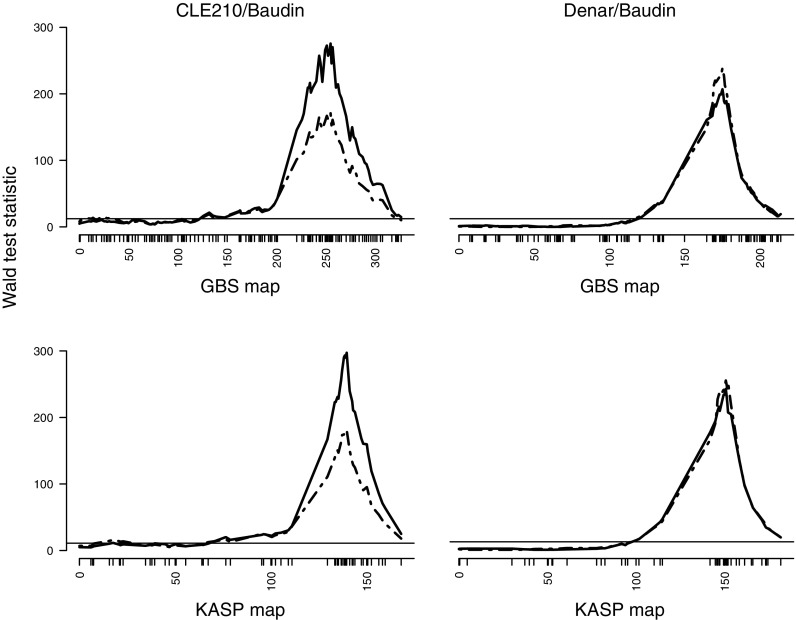
Wald test statistics for powdery mildew disease severity on adult plants in experiments conducted in 2011 (broken line) and 2012 (solid line), derived from single-marker analyses using genetic linkage maps of chromosome 5H based on genotyping by sequencing (*top*) or KASP marker genotyping (*bottom*) of the barley populations CLE210/Baudin (*left*) and Denar/Baudin (*right*). Chromosome 5H is depicted with the short arm on the *left* and the long arm on the *right*. The horizontal lines indicate the significance thresholds at α = 0.05, corrected for multiple testing

In each population, a significant QTL for adult-stage disease severity was detected on 5HL in both years (Fig. 3). For CLE210/Baudin, the QTL peaks obtained using the KASP map were considerably more distinct than those obtained using the map derived directly from GBS data. On the KASP maps, the peak test statistic values were at the positions of markers *wri184*-*wri190* (*QPm.DeBa-5H*) or markers *wri200*-*wri202* (*QPm.CLBa-5H*) (Table 2). These positions are separated by 1.0 cM in the CLE210/Baudin map and by 2.9 cM in the Denar/Baudin map (Online Resource 4).

**Table 2:** Positions and estimated effects for quantitative trait loci on chromosome 5H with significant (*p* < 0.0001) effects on powdery mildew disease severity

Population	QTL	Position (cM)	Markers	QTL effect estimate ± standard error	
				2011	2012
CLE210/Baudin	*QPm.CLBa-5H*	139.5	*wri200-wri202*	− 2.6 ± 0.2	− 2.7 ± 0.2
Denar/Baudin	*QPm.DeBa-5H*	150.7	*wri184*-*wri190*	− 2.9 ± 0.2	− 2.2 ± 0.1

These statistics were derived using genetic linkage maps of chromosome 5H derived from KASP marker genotyping of the barley populations CLE210/Baudin and Denar/Baudin. Negative QTL effect estimates indicate that the allele from the more resistant parent (CLE210 or Denar) is associated with lower disease severity than the allele from the more susceptible parent (Baudin)

Within the region of 5HL in which the QTL were mapped (between markers *wri184* and *wri204*), none of the mapped KASP markers detected polymorphism between CLE210 and Denar. With BLAST analysis of the sequences on which these markers had been designed, all but one of the markers that mapped in this region were anchored to a region of 7.6 Mbp on the 5H pseudomolecule, between 619.7 and 627.3 Mbp. Within this region, the physical order of the anchor positions exactly matches the order in which the markers had been mapped in CLE210/Baudin. To further investigate the similarity of CLE210 and Denar in this region, all of the GBS sequence tags detected in CLE210 and/or Denar were BLAST-ed against the 5H pseudomolecule. Of 65 tags that anchored in the QTL region, only three exhibited polymorphism between CLE210 and Denar and none of these genetically map on chromosome 5H.

Amongst 38 KASP markers that had been mapped near the QTL on 5HL, 36 detected the same two alleles in the cultivar panel as in the populations (Online Resource 5). The other two markers detected three alleles in the panel. One of these, *wri197*, had been mapped as a presence-absence variant (C vs. null) in the populations but detected an alternative allele (G) for some members of the panel. The other, *wri204*, had been mapped as a SNP (C vs. G) in the populations but gave a null result for some members of the panel.

Only one cultivar, Galena, exhibited the same marker haplotype as CLE210 and Denar. Three cultivars (Franklin, Hamelin and Vertess) exhibited the same haplotype as Baudin. The remaining cultivars exhibited a wide diversity of haplotypes. Amongst 139 lines for which complete data were obtained for all 38 markers, there were 77 haplotypes observed (Online Resource 6). Fifty-three cultivars had unique haplotypes that were not observed in any other line. Even the most frequent haplotype was observed in only 12 cultivars.

## Discussion

As reported by Gupta et al. (2015), CLE210 and Denar expressed resistance at the adult stage but not at the seedling stage. This resistance can be considered to be true APR. Consistent with this, of the CLE210/Baudin and Denar/Baudin DH progeny exhibited much more variation in disease severity at the adult stage than at the seedling stage and a large-effect QTL was mapped in each population for adult-stage disease severity.

With careful processing of data obtained using GBS, it was possible to construct good genetic linkage maps of all seven barley chromosomes. These maps were based mainly on SNP markers, with silicoDArT markers used only for regions in which no SNPs had been mapped. The silicoDArTs are dominant markers, scored on a presence-absence basis, with only one tag sequence reported. This limits their usefulness for both linkage mapping and marker assay design. When the ‘GBS maps’ were used to map resistance loci, it was clear that, in both CLE210 and Denar, adult-plant powdery mildew resistance is largely determined by a locus on chromosome 5H. Accordingly, sequence tags that had mapped on chromosome 5H were selected for the development of KASP assays. The resulting assays were applied to both mapping populations and a new ‘KASP map’ of chromosome 5H was constructed to replace the GBS map of that chromosome. As anticipated given the missing data and sequencing errors that are expected in GBS, better (shorter) maps were obtained using the KASP data.

The QTL peak on 5HL was much more distinct on the CLE210/Baudin KASP map than on the CLE210/Baudin GBS map. Just proximal to the QTL peak on the KASP map, there is a cluster of 13 collocating SNP markers for which the KASP data were complete and identical. Nine of these had also been mapped on the GBS map, spanning 6.4 cM. This map expansion can be attributed to spurious recombination events inferred due to incorrect genotype calls in the GBS dataset. The incorrect genotype calls would have also caused fluctuation in test statistic values. Missing data could also have affected both the map and the QTL peak. With just the GBS map, it would have been difficult to determine the peak position. In contrast, the Denar/Baudin GBS data were more complete and correct for the markers in this region. The Denar/Baudin GBS and KASP maps had the same marker order in the QTL region and had equally distinct QTL peaks.

The estimated position of the QTL on the Denar/Baudin map was similar to that on the CLE210/Baudin map, but not identical. Evaluation of all GBS tags (regardless of whether they had been retained in the curated maps) failed to reveal any evidence of sequence polymorphism between CLE210 and Denar in the 5HL QTL region. Thus, although the CLE210 and Denar QTL peaks do not exactly coincide and the two resistance sources are not known to be related, it seems likely that CLE210 and Denar are identical by descent in the QTL region and carry the same resistance allele in that region. The QTL detected here is at a similar position to QTL reported by Saghai Maroof et al. (1994), Falak et al. (1999) and Shtaya et al. (2006). However, each of these had quite minor effects, compared with the highly significant QTL reported here, and none of them were confirmed to confer true APR.

The locus mapped here could be useful for providing powdery mildew resistance in barley breeding, and some of the markers developed or evaluated here could be useful for marker-assisted selection. Amongst the markers developed and evaluated here, there is no single marker that is diagnostic of the haplotype carried by the resistance sources CLE210 and Denar. There are, however, several combinations of just a few markers that distinguish CLE210 and Denar from almost all of the other cultivars that were assayed here. One such combination consists of the four markers *wri174*, *wri182*, *wri194* and *wri206*. Individually, marker *wri174* distinguished CLE210 and Denar from over 80% of the other barley lines shown in Online Resource 6. Each of the other three markers distinguished CLE210 and Denar from over half of the other lines. Collectively, the four markers distinguished CLE210 and Denar from all but three of the barley cultivars tested: Galena, Betzes and Dobla. For any specific germplasm group, barley breeders could select markers based on the genotype information given here, then assay those markers on parental materials to verify usefulness of specific markers for particular cross combinations.

## Electronic supplementary material

Online Resource 1 (PDF 60 kb)

Online Resource 2 Frequency distributions of predicted values for powdery mildew disease severity on adult plants of CLE210/Baudin (top) and Denar/Baudin (bottom) double haploid lines, as estimated from experiments conducted in 2011 (left) and 2012 (right). Disease severity was assessed on a scale from 0 (no disease) to 9 (very severe disease). Parental predicted values and their standard errors are shown by vertical and horizontal lines, respectively (PDF 56 kb)

Online Resource 3 (PDF 13 kb)

Online Resource 4 (XLSX 20 kb)

Online Resource 5 (XLSX 50 kb)

Online Resource 6 (XLSX 29 kb)
