# Supplementary material for: A locus on barley chromosome 5H affects adult plant resistance to powdery mildew
Source: Mol Breed. 2018 Jul 28;38(8):103. doi: 10.1007/s11032-018-0858-2 (PMC6096521; doi:10.1007/s11032-018-0858-2)
Supplement: Supplementary file 1 — (PDF 60 kb) [file 11032_2018_858_MOESM1_ESM.pdf]

Article: A locus on barley chromosome 5H affects adult plant resistance to powdery mildew

Journal: Molecular Breeding

Authors: Sanjiv Gupta, Elysia Vassos, Beata Sznajder, Rebecca Fox, Kelvin H. P. Khoo, Robert Loughman, Kenneth J. Chalmers and Diane E. Mather

Corresponding author: Diane E. Mather, The University of Adelaide,

diane.mather@adelaide.edu.au

Online Resource 1

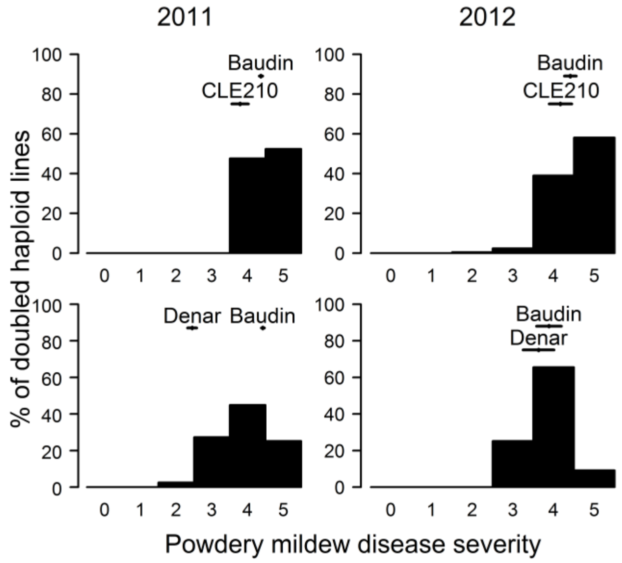

Frequency distributions of predicted values for powdery mildew disease severity on seedlings of CLE210/Baudin (top) and Denar/Baudin (bottom) double haploid lines, as estimated from experiments conducted in 2011 (left) and 2012 (right). Disease severity was assessed on a scale from 0 (no disease) to 5 (very severe disease). Parental predicted values and their standard errors are shown by vertical and horizontal lines, respectively. When the predicted values for seedling disease severity were used in QTL analysis, no QTL were detected on chromosome 5H in CLE210/Baudin. In Denar/Baudin, only a minor QTL was detected on chromosome 5H (LOD scores of 3.7 at 145.6 cM in 2011 and 5.1 at 146.8 cM in 2012, compared to LOD scores of 50.6 and 48.1, respectively, for adult plants.
