# Supplementary material for: A locus on barley chromosome 5H affects adult plant resistance to powdery mildew
Source: Mol Breed. 2018 Jul 28;38(8):103. doi: 10.1007/s11032-018-0858-2 (PMC6096521; doi:10.1007/s11032-018-0858-2)
Supplement: Supplementary file 2 — Frequency distributions of predicted values for powdery mildew disease severity on adult plants of CLE210/Baudin (top) and Denar/Baudin (bottom) double haploid lines, as estimated from experiments conducted in 2011 (left) and 2012 (right). Disease severity was assessed on a scale from 0 (no disease) to 9 (very severe disease). Parental predicted values and their standard errors are shown by vertical and horizontal lines, respectively (PDF 56 kb) [file 11032_2018_858_MOESM2_ESM.pdf]

Article: A locus on barley chromosome 5H affects adult plant resistance to powdery mildew

Journal: Molecular Breeding

Authors: Sanjiv Gupta, Elysia Vassos, Beata Sznajder, Rebecca Fox, Kelvin H. P. Khoo, Robert Loughman, Kenneth J. Chalmers and Diane E. Mather

Corresponding author: Diane E. Mather, The University of Adelaide,

diane.mather@adelaide.edu.au

Online Resource 2

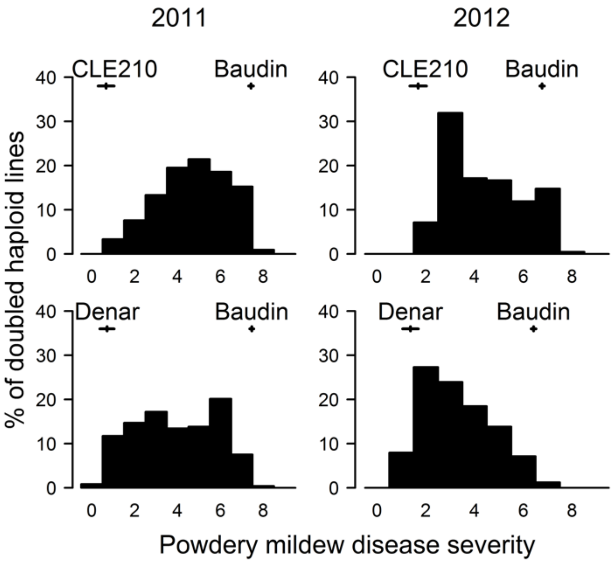

Frequency distributions of predicted values for powdery mildew disease severity on adult plants of CLE210/Baudin (top) and Denar/Baudin (bottom) double haploid lines, as estimated from experiments conducted in 2011 (left) and 2012 (right). Disease severity was assessed on a scale from 0 (no disease) to 9 (very severe disease). Parental predicted values and their standard errors are shown by vertical and horizontal lines, respectively
