# Supplementary material for: A locus on barley chromosome 5H affects adult plant resistance to powdery mildew
Source: Mol Breed. 2018 Jul 28;38(8):103. doi: 10.1007/s11032-018-0858-2 (PMC6096521; doi:10.1007/s11032-018-0858-2)
Supplement: Supplementary file 3 — (PDF 13 kb) [file 11032_2018_858_MOESM3_ESM.pdf]

Article: A locus on barley chromosome 5H affects adult plant resistance to powdery mildew

Journal: Molecular Breeding

Authors: Sanjiv Gupta, Elysia Vassos, Beata Sznajder, Rebecca Fox, Kelvin H. P. Khoo, Robert Loughman, Kenneth J. Chalmers and Diane E. Mather

Corresponding author: Diane E. Mather, The University of Adelaide, diane.mather@adelaide.edu.au

Online Resource 3

Features of curated linkage maps constructed for CLE210/Baudin and Denar/Baudin. The whole-genome maps and the GBS maps of chromosome 5H were derived from genotyping-by-sequencing data. The KASP maps were derived from KASP marker assay data for SNPs on chromosome 5H

	Whole genome		Chromosome 5H			
	CLE210/Baudin	Denar/Baudin	CLE210/Baudin		Denar/Baudin	
			GBS map	KASP map	GBS map	KASP map
Number of doubled haploid lines	200	235	200	209	235	236
Number of SNP markers	579	869	117	88	172	71
Number of silicoDArT markers	135	113	18	11	17	4
Number of positions at which markers were mapped	561	499	107	53	87	45
Total map length (cM)	1857	1372	327	168	214	183
Maximum interval length (cM)	22	32	20	19	29	27
